# Supplementary material for: The impact of city financial ecology on firm financing efficiency: Evidence from China’s strategic emerging industries
Source: PLoS One. 2023 Aug 3;18(8):e0288229. doi: 10.1371/journal.pone.0288229 (PMC10399773; doi:10.1371/journal.pone.0288229)
Supplement: Attachment — Submitted filename: Response to reviewers.docx [file pone.0288229.s002.docx]

**Rebuttal letter**

**Response to the Academic Editor**

Thank you very much for taking the time to consider my manuscript. I have tried to improve the manuscript as requested.

Kind regards,

Yong Qi

**Journal Requirements:**

1. Please ensure that your manuscript meets PLOS ONE's style requirements, including those for file naming. The PLOS ONE style templates can be found at <https://journals.plos.org/plosone/s/file?id=wjVg/PLOSOne_formatting_sample_main_body.pdf> and <https://journals.plos.org/plosone/s/file?id=ba62/PLOSOne_formatting_sample_title_authors_affiliations.pdf>

I have revised my manuscript to meet PLOS ONE's style requirements.

2. We note that the grant information you provided in the ‘Funding Information’ and ‘Financial Disclosure’ sections do not match.

When you resubmit, please ensure that you provide the correct grant numbers for the awards you received for your study in the ‘Funding Information’ section.

I have rechecked the ‘Funding Information’ and confirm that the grant numbers are correct.

3. We note that you have indicated that data from this study are available upon request. PLOS only allows data to be available upon request if there are legal or ethical restrictions on sharing data publicly. For more information on unacceptable data access restrictions, please see <http://journals.plos.org/plosone/s/data-availability#loc-unacceptable-data-access-restrictions>.

In your revised cover letter, please address the following prompts:

a) If there are ethical or legal restrictions on sharing a de-identified data set, please explain them in detail (e.g., data contain potentially sensitive information, data are owned by a third-party organization, etc.) and who has imposed them (e.g., an ethics committee). Please also provide contact information for a data access committee, ethics committee, or other institutional body to which data requests may be sent.

b) If there are no restrictions, please upload the minimal anonymized data set necessary to replicate your study findings as either Supporting Information files or to a stable, public repository and provide us with the relevant URLs, DOIs, or accession numbers. For a list of acceptable repositories, please see <http://journals.plos.org/plosone/s/data-availability#loc-recommended-repositories>.

We will update your Data Availability statement on your behalf to reflect the information you provide.

I have uploaded the data set as “S1_Dataset.xlsx” and marked it in the section "Supporting information".

4. We note that Figure 1 in your submission contain [map/satellite] images which may be copyrighted. All PLOS content is published under the Creative Commons Attribution License (CC BY 4.0), which means that the manuscript, images, and Supporting Information files will be freely available online, and any third party is permitted to access, download, copy, distribute, and use these materials in any way, even commercially, with proper attribution. For these reasons, we cannot publish previously copyrighted maps or satellite images created using proprietary data, such as Google software (Google Maps, Street View, and Earth). For more information, see our copyright guidelines: <http://journals.plos.org/plosone/s/licenses-and-copyright>.

We require you to either (1) present written permission from the copyright holder to publish these figures specifically under the CC BY 4.0 license, or (2) remove the figures from your submission:

a. You may seek permission from the original copyright holder of Figure 1 to publish the content specifically under the CC BY 4.0 license.

We recommend that you contact the original copyright holder with the Content Permission Form (<http://journals.plos.org/plosone/s/file?id=7c09/content-permission-form.pdf>) and the following text:

“I request permission for the open-access journal PLOS ONE to publish XXX under the Creative Commons Attribution License (CCAL) CC BY 4.0 (<http://creativecommons.org/licenses/by/4.0/>). Please be aware that this license allows unrestricted use and distribution, even commercially, by third parties. Please reply and provide explicit written permission to publish XXX under a CC BY license and complete the attached form.”

Please upload the completed Content Permission Form or other proof of granted permissions as an "Other" file with your submission.

In the figure caption of the copyrighted figure, please include the following text: “Reprinted from [ref] under a CC BY license, with permission from [name of publisher], original copyright [original copyright year].”

b. If you are unable to obtain permission from the original copyright holder to publish these figures under the CC BY 4.0 license or if the copyright holder’s requirements are incompatible with the CC BY 4.0 license, please either i) remove the figure or ii) supply a replacement figure that complies with the CC BY 4.0 license. Please check copyright information on all replacement figures and update the figure caption with source information. If applicable, please specify in the figure caption text when a figure is similar but not identical to the original image and is therefore for illustrative purposes only.

The following resources for replacing copyrighted map figures may be helpful:

USGS National Map Viewer (public domain): <http://viewer.nationalmap.gov/viewer/>

The Gateway to Astronaut Photography of Earth (public domain): <http://eol.jsc.nasa.gov/sseop/clickmap/>

Maps at the CIA (public domain): <https://www.cia.gov/library/publications/the-world-factbook/index.html> and <https://www.cia.gov/library/publications/cia-maps-publications/index.html>

NASA Earth Observatory (public domain): <http://earthobservatory.nasa.gov/>

Landsat: <http://landsat.visibleearth.nasa.gov/>

USGS EROS (Earth Resources Observatory and Science (EROS) Center) (public domain): [http://eros.usgs.gov/#](http://eros.usgs.gov/)

Natural Earth (public domain): <http://www.naturalearthdata.com/>

I have removed the figure and presented the results in table format instead. Please see “Table 4. Score distribution of city financial ecology”.

5. Please review your reference list to ensure that it is complete and correct. If you have cited papers that have been retracted, please include the rationale for doing so in the manuscript text, or remove these references and replace them with relevant current references. Any changes to the reference list should be mentioned in the rebuttal letter that accompanies your revised manuscript. If you need to cite a retracted article, indicate the article’s retracted status in the References list and also include a citation and full reference for the retraction notice.

I added 5 references in my revised manuscript as blow:

1. Ullah A, Zhao X, Kamal M A, Riaz A, Zheng B. Exploring asymmetric relationship between Islamic banking development and economic growth in Pakistan: Fresh evidence from a non‐linear ARDL approach. International Journal of Finance & Economics, 2021; 26(4): 6168-6187.
2. Ullah A, Zhao X, Kamal M A, Zheng J. Modeling the relationship between military spending and stock market development (a) symmetrically in China: An empirical analysis via the NARDL approach. Physica A: Statistical Mechanics and its Applications, 2020; 554: 124106.
3. Ren Z. Research on the efficiency of financial support for strategic emerging industries: Evidence from listed companies in seven major industries. Communication of Finance and Accounting, 2021; (4): 160-163.
4. Hu J. Study on heterogeneity and financing pattern matching of strategic emerging industries ——Panel data of 120 listed companies in strategic emerging industries. Journal of Social Sciences, 2020; (4):44-57.
5. Ma J, Wang J. On efficiency of financial support for strategic emerging industries ——Based on empirical analysis of the Yangtze River. Forum on Science and Technology in China, 2019; (10): 52-58.

Also, I have updated my reference list to ensure that it is complete and correct.

**Response to Reviewers**

Reviewer #1

Thank you very much for your valuable comments. I have placed my clariﬁcations and declared the corresponding changes to the manuscript after each of your points below.

I have reviewed the manuscript titled “The impact of city financial ecology on firm financing efficiency: Evidence from Chinese strategic emerging industries”

Upon thorough observations I came across the following discrepancies in the manuscript and the authors should incorporate the following suggestions with due consideration.

1) Policy Implication statement is missing in the abstract.

I have added policy implication statement to the abstract in my revised manuscript.

2) Highlight whether the proposed econometric approach is best one matching with the economic question addressed in this paper. Try to put out this issue in the introduction.

I have done this work in my revised manuscript, and put the contents in the section “Methodology”.

3) Please explicitly elaborate on how this research work contributes to the extant literature or what facets make it novel.

I have done this work in my revised manuscript. Please see the section “Introduction”.

4) Highlight the importance of financial institutions in uplifting the economy as such variables propels economic growth via difference supply and demand channels. The following articles may help them in this regard.

<https://doi.org/10.1002/ijfe.2115>

<https://doi.org/10.1016/j.physa.2019.124106>

The articles you recommended have greatly inspired me, and I have cited them in the section "Theoretical analysis " to further strengthen the theoretical support for the classification of "financial ecology growth" by highlighting the importance of financial institutions in uplifting the economy.

5) Descriptive statistics is missing. Please report it.

I have done this work in my revised manuscript. Please see the section “Results and discussion”.

6) The discussion section weak. Please back up your work with the existing pertinent literature.

I have strengthened the discussion section in my revised manuscript.

7) The conclusion section should be improved based on the study's findings. The authors also need to mention the study limitations and future research directions.

I have done these works in my revised manuscript. Please see the section “Conclusion and policy implication” and “Limitations and future works”.

Reviewer #2

Thank you very much for the time invested and for the valuable comments.

Article is well prepared and formatted accordingly. tables and equations are formatted according to the guidelines. However, there is minor suggestion to recheck headings and text formatting and spacing following the tables in the manuscript.

I have rechecked and revised them to meet PLOS ONE's style requirements.
